# Non-alcoholic fatty liver disease in a pediatric patient with heterozygous familial hypobetalipoproteinemia due to a novel *APOB* variant: a case report and systematic literature review

**DOI:** 10.3389/fmed.2023.1106441

**Published:** 2023-06-13

**Authors:** Neza Molk, Mojca Bitenc, Darja Urlep, Mojca Zerjav Tansek, Sara Bertok, Katarina Trebusak Podkrajsek, Ursa Sustar, Jernej Kovac, Tadej Battelino, Marusa Debeljak, Urh Groselj

**Affiliations:** ^1^Department of Pediatric Endocrinology, Diabetes and Metabolism, University Medical Center-University Children's Hospital, Ljubljana, Slovenia; ^2^Faculty of Medicine, University of Ljubljana, Ljubljana, Slovenia; ^3^Department of Gastroenterology, Hepatology and Nutrition, University Children's Hospital Ljubljana, University Medical Center, Ljubljana, Slovenia; ^4^Clinical Institute for Special Laboratory Diagnostics, University Children's Hospital, University Medical Center Ljubljana, Ljubljana, Slovenia

**Keywords:** *APOB*, *APOB* gene, familial hypobetalipoproteinemia, nonalcoholic steatohepatitis, hypocholesterolemia, pediatric, fatty liver, systematic review

## Abstract

**Background:**

Familial hypobetalipoproteinemia (FHBL) is an autosomal semi-dominant disorder usually caused by variants in the *APOB* gene that frequently interferes with protein length. Clinical manifestations include malabsorption, non-alcoholic fatty liver disease, low levels of lipid-soluble vitamins, and neurological, endocrine, and hematological dysfunction.

**Methods:**

Genomic DNA was isolated from the blood samples of the pediatric patient with hypocholesterolemia and his parents and brother. Next-generation sequencing (NGS) was performed, and an expanded dyslipidemia panel was employed for genetic analysis. In addition, a systematic review of the literature on FHBL heterozygous patients was performed.

**Case report:**

Genetic investigation revealed the presence of a heterozygous variant in the *APOB* (NM_000384.3) gene c.6624dup[=], which changes the open reading frame and leads to early termination of translation into the p.Leu2209IlefsTer5 protein (NP_000375.3). The identified variant was not previously reported. Familial segregation analysis confirmed the variant in the mother of the subject, who also has a low level of low-density lipoprotein and non-alcoholic fatty liver disease. We have introduced therapy that includes limiting fats in the diet and adding lipid-soluble vitamins E, A, K, and D and calcium carbonate. We reported 35 individuals with *APOB* gene variations linked to FHBL in the systematic review.

**Conclusion:**

We have identified a novel pathogenic variant in the *APOB* gene causing FHBL in pediatric patients with hypocholesterolemia and fatty liver disease. This case illustrates the importance of genetic testing for dyslipidemias in patients with significant decreases in plasma cholesterol as we can avoid damaging neurological and ophthalmological effects by sufficient vitamin supplementation and regular follow-ups.

## 1. Introduction

Hypobetalipoproteinemias (HBLs) encompass a diverse range of disorders that are characterized by reduced levels of total cholesterol (TC), low-density lipoprotein-cholesterol (LDL-C), and apolipoprotein B (apoB) in the blood, which fall below the 5th percentile of the population distribution (1).

It may develop due to primary and secondary causes (1). Primary hypobetalipoproteinemia comprises various genetic conditions, such as abetalipoproteinemia (ABL) (genetic defect in *MTP* gene), chylomicron retention disease (genetic defect in *SAR1B* gene), and combined familial hypolipidemia (genetic defect in *ANGPTL3* gene). These disorders share an autosomal recessive mode of inheritance. Another condition falling under primary hypobetalipoproteinemias is familial hypobetalipoproteinemia (FHBL) (2). The main secondary causes are malnutrition, intestinal fat malabsorption, severe liver disease, and hyperthyroidism (3).

Familial hypobetalipoproteinemia has an autosomal semi-dominant pattern of inheritance (4). Variants in the *APOB* gene are the main cause of FHBL. Additionally, less frequent variants in the *PCSK9* gene have also been associated with the disease (5, 6). Most variants in the *APOB* gene cause the formation of truncated forms of apoB, which depending on the length may or may not be secreted into the plasma (7, 8). LDL-C levels in heterozygous FHBL are low but not absent (9). Due to the lower production rate, predicted apoB levels in FHBL heterozygotes would be 50% of normal; however, they are ~24% (10). Homozygous FHBL individuals commonly exhibit significantly low levels of total cholesterol (TC) in their bloodstream, with the presence of plasma apoB either completely absent or found only in minimal quantities (2).

We report a new heterozygous variant in the *APOB* gene identified in a patient whose symptoms were characteristic of FHBL. This case prompted us to carry out a systematic review to better define the genetic causes, already identified variants, and the clinical course in heterozygous FHBL.

## 2. Methods

### 2.1. Study design and family description

The patient has been followed regularly at the University Medical Centre Ljubljana. The medical records were used to acquire his clinical information. This report is a part of the research project approved by the National Medical Ethics Committee of Slovenia (#0120-273/2019/9). The principles of the Declaration of Helsinki were followed. Written informed consent using local consent forms was obtained from the parents of the patient for the publication of any data included in this article.

### 2.2. Liver enzymes, lipid profile, and fat-soluble vitamins analysis

Laboratory measurements were analyzed for lipids including TC, LDL-C, high-density lipoprotein cholesterol (HDL-C), and triglycerides (TG) using the direct enzymatic colorimetric method. Additionally, liver enzymes, including aspartate transaminase (AST) and alanine transaminase (ALT), and fat-soluble vitamins E, A, and D were measured.

### 2.3. Genetic analysis

Genetic analysis was performed at the University Children's Hospital Ljubljana in Slovenia. Using the FlexiGene DNA Kit 250, genomic DNA was extracted from peripheral blood samples (Qiagen, Hilden, Germany). Next-generation sequencing (NGS) was performed. Using TruSight One, Illumina, FC-14111006 (Illumina, San Diego, CA, United States), the regions of interest were enriched. Sequencing was performed on the MiSeq desktop sequencer together with MiSwq reagent kit v3 (Illumina, San Diego, CA, United States). Coding regions of 4,813 genes were analyzed. A panel of seven genes associated with hypocholesterolemia, including the *APOB* gene, was used for filtering variants. VarAFT tool was applied for annotation and filtration (11). The detected variants were classified according to the American College of Medical Genetics and Genomics and the Association for Molecular Pathology (ACMG AMP) (12) classification criteria as (likely) benign, variants of uncertain significance (VUS), and (likely) pathogenic. The variant identified in the *APOB* gene was subsequently confirmed by Sanger sequencing.

### 2.4. Systematic literature review

We have collected accessible scientific report publications for the systemic review. A systematic literature review was performed on 20 June 2022, following PRISMA reporting guidelines. We searched the PubMed database for available case report articles on heterozygous patients with pathogenic and likely pathogenic variants in the *APOB* gene related to FHBL. The following search terms were used: “hypocholesterolemia” and “APOB”. In addition to that, we also searched Human Gene Variant Database Professional (13) and the Franklin by Genoox tool (14) based on the variant confirmed in our patient and using scope “Gene.” We found 63 articles. We read the abstracts and included all articles that (1) were published in English, (2) were fully accessible, (3) contained human data and clinical data on patients, and (4) described heterozygous cases. We excluded all articles that did not meet the criteria. All articles that met the criteria were read and analyzed in full-text form. In the end, 19 articles describing 35 cases were included in our systematic literature review. All data were collected in Microsoft Office 365 (Microsoft Corporation, Redmond, WA, USA). Systematic literature workflow is presented in Figure 4.

## 3. Case report

The proband was a male in his early twenties who was referred to our department for HBL at the age of 10 years. He was hospitalized in another hospital for gastroenterocolitis at the age of 7 years, where laboratory tests revealed severe hypobetalipoproteinemia. The family history revealed that his two siblings as well as his mother and his maternal grandmother all have low serum levels of LDL-C. His mother and sister have been diagnosed with celiac disease.

**Figure 1:**
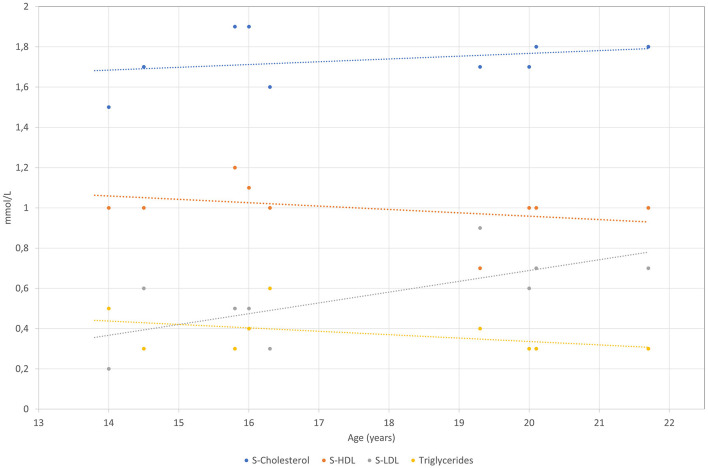
S-Cholesterol, S-HDL-C, S-LDL-C and S-TAG levels are presented over time for the patient measured in mmol/L.

**Figure 2:**
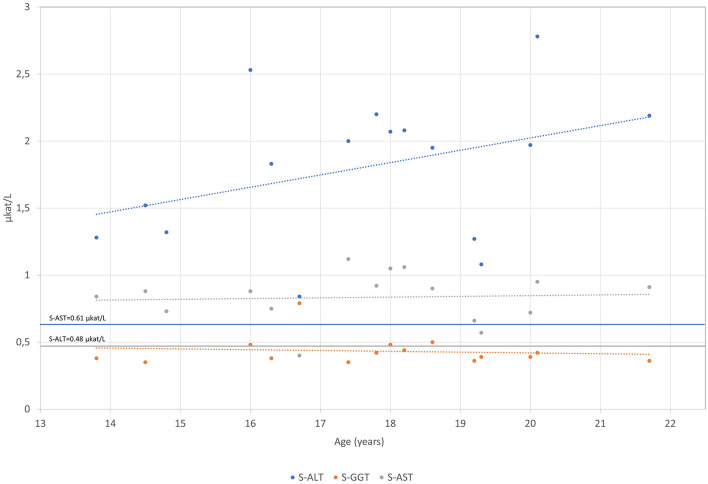
S-AST, S-ALT and S-GGT levels are presented over time for the patient measured in μkat/L.

**Figure 3:**
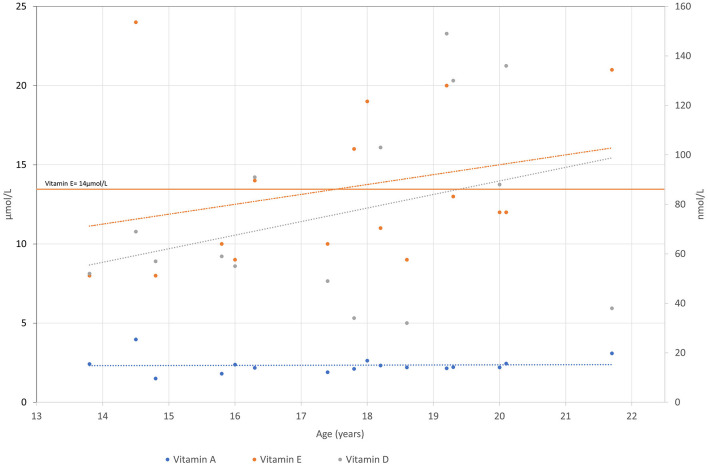
Vitamin A, E and D levels are presented over time for the patient. Vitamin A and E are measured in μkat/L and vitamin D in nmol/L.

**Figure 4:**
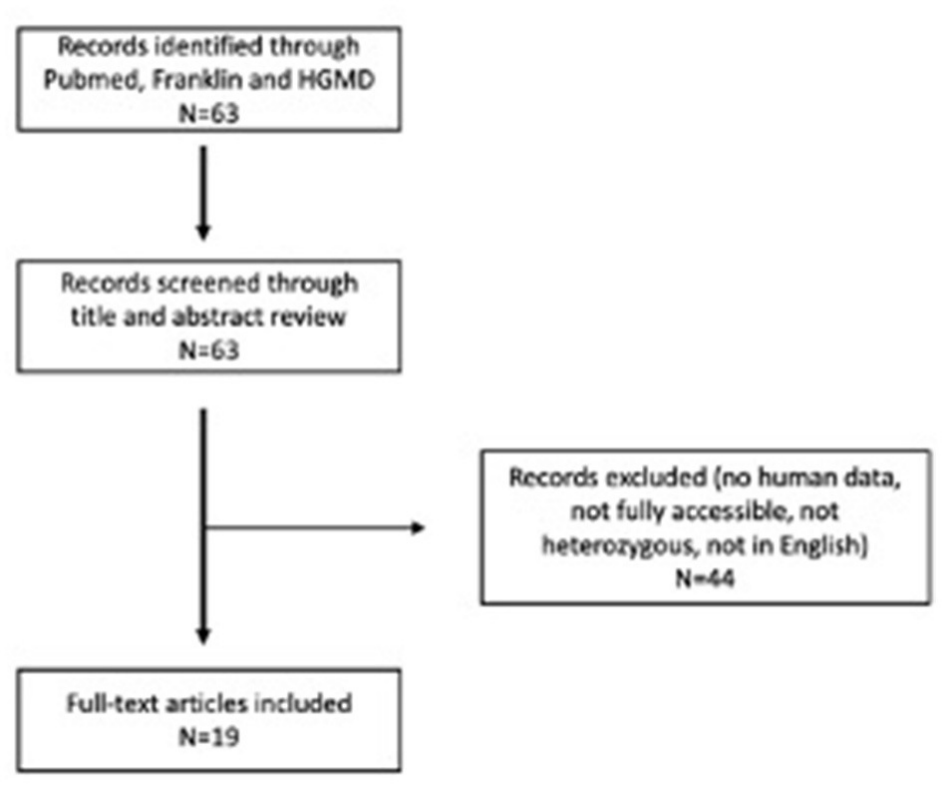
PRISMA flow diagram for systematic literature review.

The patient was born after a fourth pregnancy, the second pregnancy ended with a miscarriage. He has had atopic dermatitis since birth and in the last years, allergic rhinitis has developed. The patient also reported getting tired very quickly, with unpleasant breath in the morning. On physical examination, he was quite high for his age with a body mass index of 17.5 kg/m^2^. Respiratory and cardiovascular examinations were normal. His abdomen was a little bit tender with a palpable liver of 2 cm under the rib cage and impalpable other organs or masses. Other physical findings included an asymmetric chest and irregular growth of teeth. After examination, several blood tests were performed, revealing mildly elevated levels of AST 54 IU/L (0.9 μkat/L) (<36.6 IU/L, <0.61 μkat/L) and ALT 100.8 IU/L (1.68 μkat/L) (<28.8 IU/L, <0.48 μkat/L) with normal gamma-glutamyl transferase (gGT) and bilirubin concentrations. Low levels of LDL-C of 0.4 mmol/L (2.0–3.5 mmol/L) and apoB of 0.2 (0.55–1.40 mmol/L) were found. Hormones and antibodies regarding any thyroid gland disorder were in a normal range. Coeliac disease was excluded. The proband was afterward regularly followed by our endocrinology department and by a gastroenterologist. Laboratory tests are chronologically presented in Figures 1–4.

The mildly elevated ALT and AST persisted until the end of the follow-up at our tertiary center. The concentration of ALT varied from 63.5 to 163.5 IU/L (1.08–2.78 μkat/L) and AST from 33.5 to 55.9 IU/L (0.57 to 0.95 μkat/L) (Figure 2). The concentrations of gGT and bilirubin were within normal limits. Abdominal ultrasound revealed a marginally enlarged liver and spleen (at the upper limit for age and gender). The gallbladder and bile ducts appeared normal. Other abdominal organs were without evident pathology. Magnetic resonance of the upper abdomen showed changes characteristic of hepatic steatosis. A liver biopsy was performed at the age of 16 years, which confirmed liver steatosis (steatosis was present in 50% of hepatocytes). There were no signs of liver cell injury (e.g., ballooning degeneration) or inflammation; however, there was evidence of mild fibrosis along the central veins and mild sinusoidal-pericellular fibrosis. Other causes of chronic liver diseases were excluded (e.g., viral liver disease, Wilson's disease, alpha-1-antitrypsin deficiency, autoimmune liver disease, hemochromatosis, drug-induced liver steatosis, and others). During the follow-up, the synthetic liver function was normal. There were no signs of portal hypertension or hypersplenism. The patient was without gastrointestinal symptoms throughout the follow-up, and he passed normally formed stools once or twice a day. Serological tests for coeliac disease were repeated several times and were always negative. He was not nourished, and his body mass index (BMI) ranged between 17.5 kg/m^2^ at the diagnosis of hypobetalipoproteinemia and 24.2 kg/m^2^ at the last follow-up examination at the age of 18 years. At that time, the values of ALT and AST were 74.7 IU/L (1.27 μkat/L) and 38.8 IU/L (0.66 μkat/L) respectively, with normal gGT and bilirubin concentrations, and there were no signs of portal hypertension on Doppler ultrasound of the portal veins.

Low levels of vitamin E were one of the major concerns regarding our proband. The blood test of proband aged 10 years revealed vitamin E levels of 13.6 μmol/L (14–23 μmol/L). He was prescribed a treatment of 10 mg of vitamin E per day and 1,000 IE of vitamin D per day. He was also treated with lecithin, complex B, and omega-3 fatty acids. As the measured vitamin E level did not improve, the dose was initially increased to 100 mg per day and later gradually to 1,000 mg per day to achieve adequate blood concentrations.

The first bone densitometry performed in 2011 was unremarkable (L1–L4 Z-score +0.3; whole body Z-score −0.3). He was prescribed calcium carbonate 1,000 mg per day and 1,000 IE of vitamin D per day due to osteopenia that was revealed on the second densitometry (L1–L4: Z-score −0.5; whole body Z-score −1.6).

During the follow-up, he was regularly followed by a clinical dietician. At the start of the follow-up, an analysis of the dietary diary revealed that the proband's intake was 2,000 kcal/day (recommended 3,000 kcal/day), fat intake was as recommended (<30% of total calories), but with long-chain fatty acids around 30 g/day, whereas 15 g/day are recommended for the patients with the HBL.

**Figure 5:**
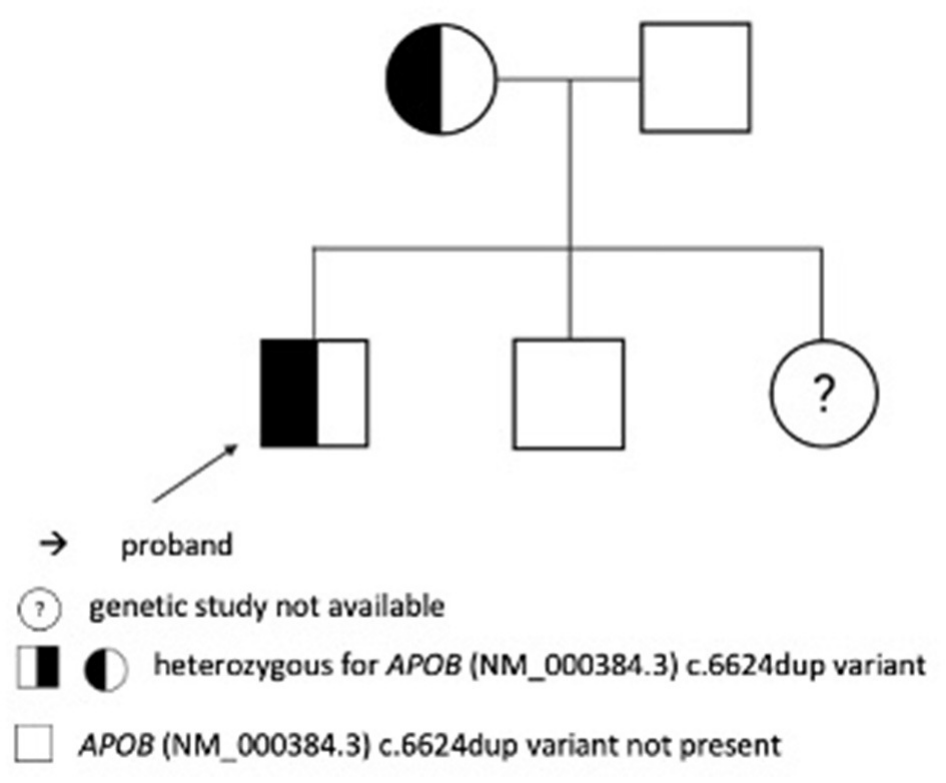
A family pedigree with c.6624dup p.Leu2209IlefsTer5 variant in the APOB gene.

According to the following US of the abdomen in 2017, improvement was noticed. A liver biopsy revealed histologic changes in line with metabolic liver defects. It showed microvesicular steatosis in 50% of hepatocytes without steatohepatitis, fibrosis along the central veins, and mild sinusoidal-pericellular fibrosis. The first genetic analysis was performed in 2012. Exon 26 of the *APOB* gene was amplified by a polymerase chain reaction and sequenced directly to analyze the presence of variants NM_000384.3:c.10707C>T and NM_000384.3:c.10708G>A. Analyzed variants were not confirmed, thus FHBL could not be confirmed as the cause for the clinical manifestation of the disease. The diagnosis was confirmed after a second genetic analysis was performed using NGS. In the *APOB* (OMIM: +107730) gene, heterozygous variant c.6624dup has been identified, which we then confirmed with Sanger sequencing. The variant is not found in dbSNP, HGMD, or gnomAD databases. The *in silico* prediction tools report variants as pathogenic (CADD (https://cadd.gs.washington.edu/), Variant Taster). The variant is classified as pathogenic (PVS1 very strong, PM2 moderate, and PP1 supporting) according to the ACMG-AMP criteria (12). Thus, this variant was considered causal for the clinical manifestations. Later, Sanger sequencing-based segregation analysis confirmed the presence of this variant in proband's mother, but it was not confirmed in proband's brother and father. A family peedigree with c.6624dup p.Leu2209IlefsTer5 variant in the APOB gene is presented in Figure 5. We were unable to perform a genetic analysis on his sister who has normal cholesterol levels. In 2019, we expended genetic testing which revealed the variant in the *COL2A1* gene (NM_001844.5) c.375+1G>A[=], confirming the Stickler syndrome.

In the next follow-ups in 2018 and 2019, treatment and diet remained the same. The US of the abdomen in 2019 showed regression of the steatohepatitis compared with the previous US of the abdomen at the beginning of the same year, which showed moderate hepatosplenomegaly and hepatopathy. Vitamin E level was still at the lower limit of the normal range, and the supplementation dose was increased to 2,000 mg/day. The last follow-up visit was in 2022. Apart from elevated liver enzymes with AST 53.5 IU/L (0.91 μkat/L) (<35.9 IU/L, <0.61 μkat/L) and ALT 128.8 IU/L (2.19 μkat/L) (<28.2 IU/L, <0.48 μkat/L), everything else remained stable and treatment remained unchanged. All biochemical measurements are collected and presented in Supplementary Table 1 and on the Mendeley Data Repository https://doi.org/10.17632/sm84p6vp26.1.

## 4. Discussion

Our study presented the results of a follow-up conducted over an extended period for a proband carrying a new variant in the *APOB* gene, which is associated with FHBL.

FHBL type 1 (OMIM: #615558) is a semi-dominant disorder mainly caused by protein-truncating variants (PTVs) in the *APOB* gene (15). In 1979, Steinberg and his colleagues were the first to identify a distinct condition characterized by hypobetalipoproteinemia and normal triglyceridemia. Young et al. described the first kindred with two distinct abnormal *APOB* alleles associated with FHBL (16). Multiple following kindreds have been documented since then, which has allowed the identification of numerous gene deletions, single nucleotide substitutions, and splicing variants.

The *APOB* gene, positioned on chromosome 2p24.1, is composed of 29 exons. The same gene produces two forms of protein, apoB-48 and apoB-100. ApoB-48 is produced by tissue-specific mRNA C → U RNA editing at nucleotide position 6666 which causes premature stop codon. The modification results in the production of apoB-48, representing 48% of the amino terminus of apoB-100 (17). ApoB-48 is produced by the small intestine and lacks the LDL-receptor binding domain, but it plays a crucial role in chylomicron synthesis (18, 19). Full-length apoB-100 is produced in the liver. It plays a role as a structural component of very low-density lipoprotein (VLDL) and a ligand for receptor-mediated endocytosis of low-density lipoprotein (LDL).

Due to their enormous size and hydrophobicity, apoB-100 and apoB-48's 3D structures have not yet been fully characterized at the anatomic level. However, attempts have been made to determine the structure of the apoB-100 domains using different algorithms (20). The modeled apoB-100 protein is composed of five domains NH3-βα1-β1-α2-β2-α3-COOH. The initial assembly of triglyceride-rich lipoproteins in enterocytes and hepatocytes depends on the N-terminal βα1 superdomain (N-terminal amino acids 1–827), which has both α-helical and β-sheet secondary structures. β1 domain, which is located roughly between amino residues 827 and 2000, is involved in irreversible lipid-binding, while the β2 provides LDL receptor-binding abilities (21). The α2 domain, located roughly between amino residues 2,075 and 2,575, and the α3 domain, located roughly between amino residues 4,100 and 4,550, form amphipathic α -helices with both hydrophilic and hydrophobic characteristics. They provide a flexible area that gives the molecule elasticity and enables the recruitment of varying amounts of core lipids (22). ApoB-48 is composed of the domains βα1, β1, and a portion of the domain α2 (20).

The majority of the FHBL-causing variants identified interfere with the development of a complete apoB-100 molecule (19). The size and density of lipoprotein particles depend on the length of apoB truncation. Patients with variants causing truncated proteins have lower plasma levels of apoB possibly because of lower apoB production and higher clearance rate (23). The ability of apoB to create plasma lipoproteins in the liver or intestine and export lipids from these organs is lost when apoB-48 is in an inadequate form (24). The severity of symptoms is related to truncation lengths; long-truncated apoBs continue to have some lipid-binding ability, whereas truncations that are shorter than apoB-29/30 are primarily degraded within cells and are not released as part of lipoprotein particles. Truncations shorter than apoB-29/30 lead to a phenotype similar to ABL, characterized by neurologic dysfunctions, fatty liver, acanthocytosis, a lack of fat-soluble vitamins, and fat malabsorption (1, 25). Carriers with longer truncations are less affected (e.g., longer than apoB-75) as they still have some capacity to bind lipids and form lipoprotein particles that are then released into the bloodstream. However, in the presence of certain factors, such as a high-fat diet, alcohol consumption, or obesity, individuals with longer truncated apoBs may still develop fatty liver. Most people with heterozygous FHBL do not experience any symptoms but may have mild liver dysfunction and hepatic steatosis. However, around 5–10% of them may develop more severe non-alcoholic steatohepatitis that requires medical treatment and, in rare cases, may progress to cirrhosis (26). Other symptoms observed in heterozygous patients include increased stool frequency, chronic steatorrhea, mild vitamin deficiencies, and malabsorption, particularly after consuming high-fat meals (27).

The clinical severity of FHBL in individuals who are homozygous or compound heterozygous for apoB truncations is determined by the ability of truncated apoBs to bind lipids and form lipoprotein particles. FHBL homozygotes or compound heterozygotes carrying apoB truncations, that result in both alleles encoding truncated apoBs shorter than ApoB-29/30, do not have detectable apoB in their bloodstream. For individuals who are homozygous or compound heterozygous for truncations longer than apoB-50, the clinical phenotype can vary widely. In severe cases, it may lead to neurological complications due to the malabsorption of vitamin E (28). The malabsorption of fat can be managed through dietary modifications, such as limiting the consumption of long-chain fatty acids, and supplementing fat-soluble vitamins (1).

In Supplementary Table 2, we conducted a literature review on the variants in the *APOB* gene that have been observed in heterozygous FHBL patients so far. Our review includes 19 articles describing 35 cases. The most common type of variants observed are substitutions in exons (43%). Truncation lengths vary from 6.46–83% of the full apoB-100 length. Among the reported symptoms, fatty liver was described in 57% of cases, and it was also exhibited in our proband.

We report a novel single nucleotide duplication [NM_000384.3:c.6624dup[=]] in exon 26 of the *APOB* gene, which was found in a family with HBL. This variant causes a frameshift in translation p.Leu2209IlefsTer5 protein (NP_000375.3), early termination, and results in a truncated protein. In our case, frameshift causes termination codon TGA at the 2213th amino acid. This represents 48.5% of full apoB length or intermediate size truncation which is longer than the wild-type counterpart. This variant is located in α2 based on protein structure. Previous studies have shown that individuals who are FHBL heterozygotes and carry truncated apoBs have lower-than-expected levels of plasma LDL-C and apoB, which are approximately one-third of normal levels. This is due to a combination of reduced hepatic secretion of apoB-100, increased catabolism of VLDL, and decreased secretion of the truncated apoBs (29). Martín-Morales et al. presented a heterozygous case with the Ser2184fsVal2193X variant that produces a protein apoB-48.32 (a truncated apoB comparable in size to that found in our kindred) (2013). Like our proband, their case also showed symptoms of steatorrhea, fatty liver, and low concentrations of TC, LDL-C, and apoB. In addition, we also discovered the variant in the *COL2A1* gene (NM_001844.5) c.375+1G>A[=], confirming the Stickler syndrome. Stickler syndrome affects connective tissue and can involve multiple systems in the body, including the inner ear, eyes, joints, and skeleton. The disorder is characterized by a range of symptoms, including myopia (nearsightedness), vitreoretinal degeneration, cataracts, and retinal detachment at a young age (30). These eye manifestations were also observed in the case being discussed. Since some of FHBL and Stickler syndrome symptoms overlap, we cannot solely attribute all the characteristics observed in our proband to the variant discovered in the *APOB* gene.

According to previous studies, patients with FHBL have been shown to have lower levels of lipid-soluble vitamins E, A, K, and D in their cellular membranes. Vitamin A plays a crucial role in phototransduction and vitamin E is important for normal retinal function. Vitamin E transport is hindered in FHBL due to the fact that it heavily relies on transport through chylomicrons and other particles that contain apo B, which are impaired in FHBL (8). Supplementation of vitamin E can prevent the onset of retinopathy, or if it has already developed, it can halt its progression (31). Homozygous FHBL patients may present neurological and ophthalmological abnormalities, which include progressive spinocerebellar degeneration, areflexia, ataxia, and retinitis pigmentosa due to lipid-soluble vitamins deficiency (4). They usually experience neurological symptoms in their first or second decade of life. Early identification of these conditions through an effective screening program is crucial, as proper treatment can prevent adverse neurological and ophthalmological outcomes (2).

The current standard treatment involves dietary restrictions and vitamin supplementation. It is recommended to consider supplementation when low levels of lipid-soluble vitamins are detected. Vitamin E doses should be close to the recommended daily intake (15 mg/day), with tolerable upper intake levels of 1,000 mg/day. In our case, our patient was prescribed up to 2,000 mg/day due to irregular intake. To prevent ophthalmologic complications, high doses of vitamin A (100–400 IU/kg/day) should be given if a deficiency is detected. Additionally, it is recommended to supplement vitamin D (800–1,200 IU/day) in all patients. Due to malabsorption, it is recommended to reduce long-chain fatty acids in the diet. Regular clinical judgment is recommended for all homozygous patients and heterozygous patients with vitamin deficiencies and fatty liver disease. Periodic transaminase tests and abdominal ultrasound can be used to monitor hepatic function. Follow-up evaluations for older patients include ophthalmologic assessments to detect atypical retinitis pigmentosa and tests to measure bone mineral density to check for osteopenia (2). Our patient was managed with vitamin E, vitamin D3, calcium carbonate, lecithin, complex B, and omega-3 fatty acids. Doses were adjusted based on the deficiency.

In conclusion, early detection and treatment of patients with FHBL are of great importance since damaging neurological and ophthalmological effects may be avoided by following up with sufficient vitamin supplementation. Early detection is possible through genetic testing of subjects showing symptoms of malabsorption and a decrease in plasma cholesterol or, ideally, through a screening program for familial hypercholesterolemia (32–35). Our proband had a novel heterozygous *APOB* variant causing significant liver steatosis, malabsorption of fat-soluble vitamins, and osteopenia. Treatment with sufficient vitamins and an adequate diet helped at limiting disease symptoms progression (36).

## Data availability statement

The datasets for this article are not publicly available due to concerns regarding participant/patient anonymity. Requests to access the datasets should be directed to the corresponding author.

## Ethics statement

The studies involving human participants were reviewed and approved by the National Medical Ethics Committee (#0120-273/2019/9). Written informed consent to participate in this study was provided by the participants' legal guardian/next of kin.

## Author contributions

NM and MB collected the data and prepared the first draft of the manuscript. MZ, DU, and UG followed and treated the patient, performed follow-up tests, and collected the data. MD, SB, US, and JK performed the molecular genetic analysis and data analysis with interpretation. UG supervised the work and helped write the manuscript. All authors approved the final manuscript as submitted and agreed to be accountable for all aspects of the work.
