# Supplementary material for: Non-alcoholic fatty liver disease in a pediatric patient with heterozygous familial hypobetalipoproteinemia due to a novel APOB variant: a case report and systematic literature review
Source: Front Med (Lausanne). 2023 Jun 13;10:1106441. doi: 10.3389/fmed.2023.1106441 (PMC10293746; doi:10.3389/fmed.2023.1106441)
Supplement: Supplementary file 1 [file Table_1.DOCX]

| Age (years) | Vitamin A (µmol/L) | Vitamin E (µmol/L) | Vitamin D (nmol/L) | AST (µkat/L) | ALT (µkat/L) | GGT (µkat/) | TC (mmol/L) | HDL (mmol/L) | LDL (mmol/L) | TG (mmol/L) |
| --- | --- | --- | --- | --- | --- | --- | --- | --- | --- | --- |
| 13.8 | 2.41 | 8 | 52 | 0.84 | 1.28 | 0.38 |  |  |  |  |
| 14 |  |  |  |  |  |  | 1.5 | 1 | 0.2 | 0.5 |
| 14.5 | 3.97 | 24 | 69 | 0.88 | 1.52 | 0.35 | 1.7 | 1 | 0.6 | 0.3 |
| 14.8 | 1.5 | 8 | 57 | 0.73 | 1.32 |  |  |  |  |  |
| 15.8 | 1.8 | 10 | 59 |  |  |  | 1.9 | 1.2 | 0.5 | 0.3 |
| 16 | 2.38 | 9 | 55 | 0.88 | 2.53 | 0.48 | 1.9 | 1.1 | 0.5 | 0.4 |
| 16.3 | 2.17 | 14 | 91 | 0.75 | 1.83 | 0.38 | 1.6 | 1 | 0.3 | 0.6 |
| 16.7 |  |  |  | 0.4 | 0.84 | 0.79 |  |  |  |  |
| 17.4 | 1.9 | 10 | 49 | 1.12 | 2 | 0.35 |  |  |  |  |
| 17.8 | 2.11 | 16 | 34 | 0.92 | 2.2 | 0.42 |  |  |  |  |
| 18 | 2.63 | 19 |  | 1.05 | 2.07 | 0.48 |  |  |  |  |
| 18.2 | 2.32 | 11 | 103 | 1.06 | 2.08 | 0.44 |  |  |  |  |
| 18.6 | 2.2 | 9 | 32 | 0.9 | 1.95 | 0.5 |  |  |  |  |
| 19.2 | 2.15 | 20 | 149 | 0.66 | 1.27 | 0.36 |  |  |  |  |
| 19.3 | 2.22 | 13 | 130 | 0.57 | 1.08 | 0.39 | 1.7 | 0.7 | 0.9 | 0.4 |
| 20 | 2.2 | 12 | 88 | 0.72 | 1.97 | 0.39 | 1.7 | 1 | 0.6 | 0.3 |
| 20.1 | 2.44 | 12 | 136 | 0.95 | 2.78 | 0.42 | 1.8 | 1 | 0.7 | 0.3 |
| 21.7 | 3.09 | 21 | 38 | 0.91 | 2.19 | 0.36 | 1.8 | 1 | 0.7 | 0.3 |

Supplement Table 1: Proband’s biochemical measurements
