# Supplementary material for: Non-alcoholic fatty liver disease in a pediatric patient with heterozygous familial hypobetalipoproteinemia due to a novel APOB variant: a case report and systematic literature review
Source: Front Med (Lausanne). 2023 Jun 13;10:1106441. doi: 10.3389/fmed.2023.1106441 (PMC10293746; doi:10.3389/fmed.2023.1106441)
Supplement: Supplementary file 2 [file Table_2.docx]

Supplement Table 2: Summary of the 35 identified previous cases of heterozygous *APOB* variants in familial hypobetalipoproteinemia described in 19 reports.

|  | | | | | | Symptoms present | | | | | | | | | |  |  |
| --- | --- | --- | --- | --- | --- | --- | --- | --- | --- | --- | --- | --- | --- | --- | --- | --- | --- |
| Report | Nucleotide change (NM_000384.3) | Protein change (NP_000375.3) | ACMG classification | Ancestry | Gender (M/F) | | Hepatic steatosis | Diarrhoea | Failure to thrive | Fat malabsorption | Steatorrhea | Acanthocytosis | Neurological symptoms | Asymptomatic | Other | ApoB length (% of full length) |  |
| (Talmud et al., 1994) | c.7564C>T | p.Arg2522Ter | Pathogenic | Scottish | F | |  |  |  |  |  |  |  |  | Retinitis pigmentosa | 55 |  |
|  |  |  |  |  | M | |  |  |  |  |  |  |  |  | Retinitis pigmentosa | 55 |  |
| (Musialik et al., 2020) | c.3696+1G>T |  | Likely pathogenic |  | F | | 1 |  |  |  |  |  | 1 |  |  |  |  |
|  |  |  |  |  | M | | 1 |  |  |  |  |  | 1 |  |  |  |  |
| (Welty et al., 1991) | c.9200del | p.Lys3067ArgfsTer2 | Pathogenic |  | F | |  |  |  |  |  |  |  | 1 |  | 67 |  |
|  |  |  |  |  | M | |  |  |  |  |  |  |  | 1 |  | 67 |  |
| (Farese et al., 1992) | c.11330C>A | p.Ser3777Ter | Likely pathogenic | Caucasian, Danish descent | F | |  |  |  |  |  |  |  |  | Gallstones | 83 |  |
|  |  |  |  |  | M | |  |  |  |  |  |  |  |  | Gallstones | 83 |  |
| (Di Leo et al., 2007) | c.3843-2A>G |  | Likely pathogenic |  | M | | 1 |  |  |  |  |  |  |  | Hepatomegaly |  |  |
|  |  |  |  |  | M | | 1 |  |  |  |  |  |  |  |  |  |  |
|  | c.4217-1G>T |  | Likely pathogenic |  | F | | 1 |  |  |  |  |  |  |  |  |  |  |
| (Huang et al., 1991) | c.537+1G>T |  | Pathogenic | Caucasian | M | |  |  |  |  |  |  |  | 1 |  |  |  |
|  | c.1315C>T | .Arg439Ter | Pathogenic | Caucasian | F | |  |  |  |  |  |  |  | 1 |  |  |  |
| (Wagner et al., 1991) | c.7537C>T | p.Arg2513Ter | Pathogenic | Caucasian | M | |  |  |  |  |  |  |  | 1 |  | 54,8 |  |
| (Krul et al., 1992) | c.10238del | p.Thr3413MetfsTer2 | Pathogenic |  | M | |  |  |  |  |  |  |  | 1 |  | 74,7 |  |
| (Katsuda et al., 2009) | c.1902_1903del | p.Arg635GlufsTer14 | Likely pathogenic | Japanese | F | | 1 |  |  |  |  |  |  |  |  | 13,7 |  |
| (Groenewegen et al., 1994) | c.9632dup | p.Asn3211LysfsTer14 | Likely pathogenic |  | M | |  |  |  |  |  |  |  | 1 |  |  |  |
| (Martín-Morales et al., 2013) | c.6634del | p.Asp2213MetfsTer8 | Likely pathogenic | Spanish | M | | 1 |  |  |  | 1 |  |  |  | Reduced bone mineral density | 48,32 |  |
|  | c.7600C>T | p.Arg2534Ter | Likely pathogenic | Spanish | F | | 1 |  |  |  |  |  |  |  | Down syndrome | 55,25 |  |
|  | c.11095A>T | p.Arg3699Ter | Likely pathogenic | Spanish | M | |  |  |  |  |  |  |  | 1 |  | 80,93 |  |
| (Tarugi et al., 1996) | c.5350_5363del | p.Val1784ThrfsTer12 | Likely pathogenic | Italian | M | | 1 |  |  |  |  |  |  |  |  | 38,95 |  |
| (Whitfield et al., 2003) | c.1019_1026del | p.Gln340ProfsTer4 | Likely pathogenic |  | M | | 1 |  |  |  |  |  |  |  |  | 6,9 |  |
|  | c.3600T>A | p.Tyr1200Ter | Pathogenic |  | M | | 1 |  |  |  |  |  |  |  |  | 25,8 |  |
|  | c.11040T>G | p.Tyr3680Ter | Pathogenic |  | M | | 1 |  |  |  |  |  |  |  |  | 80,5 |  |
|  | c.82+1G>A |  | Likely pathogenic |  | M | | 1 |  |  |  |  |  |  |  |  |  |  |
| (Di Leo et al., 2009) | c.3697-1G>C |  | Likely pathogenic |  | M | | 1 |  |  |  |  |  |  |  |  |  |  |
| (Lancellotti et al., 2004) | c.961C>T | p.Gln321Ter | Likely pathogenic |  | M | | 1 |  |  |  | 1 |  |  |  |  | 6,46 |  |
| (Rimbert et al., 2018) | c.2533C>T | p.Gln845Ter | Likely pathogenic |  | M | | 1 |  |  |  |  |  |  |  |  | 18 |  |
|  | c.7711C>T | p.Gln2571Ter | Likely pathogenic |  | M | | 1 |  |  |  |  |  |  |  | Pre-diabetes | 56 |  |
|  | c.11153C>G | p.Ser3718Ter | Likely pathogenic |  | M | | 1 |  |  |  |  |  |  |  | Hepatocellular carcinoma |  |  |
|  |  |  |  |  | M | | 1 |  |  |  |  |  |  |  |  |  |  |
| (Brosnahan et al., 1994) | c.7564C>T | p.Arg2522Ter | Pathogenic | USA | F | |  |  |  |  |  |  |  |  | Retinal degradation | 55 |  |
| (Tarugi et al., 2001) | c.4611T>A | p.Tyr1537Ter | Likely pathogenic |  | M | | 1 |  |  |  |  |  |  |  |  | 33,4 |  |
|  | c.10386del | p.Tyr3462Ter | Likely pathogenic |  | M | |  |  |  |  |  |  |  |  | Alcoholic steatohepatitis | 75,7 |  |
| (Collins et al., 1988) | c.3997C>T | p.Arg1333Ter | Pathogenic |  | F | |  |  |  | 1 |  |  |  |  |  |  |  |
| M= Male, F= Female | | | | | | | | | | | | | | | | |  |

35. Brosnahan DM, Kennedy SM, Converse CA, Lee WR, Hammer HM. Pathology of hereditary retinal degeneration associated with hypobetalipoproteinemia. Ophthalmology. (1994) 101:38–45. doi: 10.1016/S0161-6420(94)31358-3

36. Collins DR, Knott TJ, Pease RJ, Powell LM, Wallis SC, Robertson S, et al. Truncated variants of apolipoprotein B cause hypobetalipoproteinaemia. Nucleic Acids Res. (1988) 16:8361–75. doi: 10.1093/nar/16.17.8361

37. Di Leo E, Magnolo L, Lancellotti S, Crocè L, Visintin L, Tiribelli C, et al. Abnormal apolipoprotein B pre-mRNA splicing in patients with familial hypobetalipoproteinaemia. J Med Genet. (2007) 44:219–224. doi: 10.1136/jmg.2006.046359

38. Di Leo E, Magnolo L, Pinotti E, Martini S, Cortella I, Vitturi N, et al. Functional analysis of two novel splice site mutations of APOB gene in familial hypobetalipoproteinemia. Mol Genet Metab. (2009) 96:66–72. doi: 10.1016/j.ymgme.2008.10.016

39. Farese RV, Garg A, Pierotti VR, Vega GL, Young SG. A truncated species of apolipoprotein B, B-83, associated with hypobetalipoproteinemia. J Lipid Res. (1992) 33:569–77. doi: 10.1016/S0022-2275(20)41622-0

40. Groenewegen WA, Krul ES, Averna MR, Pulai J, Schonfeld G. Dysbetalipoproteinemia in a kindred with hypobetalipoproteinemia due to mutations in the genes for ApoB (ApoB-70.5) and ApoE (ApoE2). Arterioscl Thromb. (1994) 14:1695–704. doi: 10.1161/01.ATV.14.11.1695

41. Huang LS, Kayden H, Sokol RJ, Breslow JL. ApoB gene nonsense and splicing mutations in a compound heterozygote for familial hypobetalipoproteinemia. J Lipid Res. (1991) 32 1341–8. doi: 10.1016/S0022-2275(20)41964-9

42. Katsuda S, Kawashiri, M.-A., Inazu A, Tada H, Tsuchida M, Kaneko Y, et al. Apolipoprotein B gene mutations and fatty liver in Japanese hypobetalipoproteinemia. Clin Chim Acta Int J Clin Chem. (2009) 399:64–8. doi: 10.1016/j.cca.2008.09.021

43. Krul ES, Parhofer KG, Barrett PH, Wagner RD, Schonfeld G. ApoB-75, a truncation of apolipoprotein B associated with familial hypobetalipoproteinemia: genetic and kinetic studies. J Lipid Res. (1992) 33:1037–50. doi: 10.1016/S0022-2275(20)41419-1

44. Lancellotti S, Di Leo E, Penacchioni JY, Balli F, Viola L, Bertolini S, et al. Hypobetalipoproteinemia with an apparently recessive inheritance due to a “de novo” mutation of apolipoprotein B. Biochim Biophys Acta. (2004) 1688:61– 7. doi: 10.1016/j.bbadis.2003.11.002

45. Musialik J, Boguszewska-Chachulska A, Pojda-Wilczek D, Gorzkowska A, Szyman´czak R, Kania M, et al. A rare mutation in the APOB gene associated with neurological manifestations in familial hypobetalipoproteinemia. Int J Mol Sci. (2020) 21:1439. doi: 10.3390/ijms21041439

46. Rimbert A, Pichelin M, Lecointe S, Marrec M, Scouarnec L, Barrak E, et al. Identification of novel APOB mutations by targeted next-generation sequencing for the molecular diagnosis of familial hypobetalipoproteinemia. Atherosclerosis. (2018) 250:52–6. doi: 10.1016/j.atherosclerosis.2016.04.010

47. Talmud PJ, Krul ES, Pessah M, Gay G, Schonfeld G, Humphries SE, et al. Donor splice mutation generates a lipid-associated apolipoprotein B-27.6 in a patient with homozygous hypobetalipoproteinemia. J Lipid Res. (1994) 35:468– 77. doi: 10.1016/S0022-2275(20)41197-6

48. Tarugi P, Lonardo A, Ballarini G, Grisendi A, Pulvirenti M, Bagni A, et al. Fatty liver in heterozygous hypobetalipoproteinemia caused by a novel truncated form of apolipoprotein B. Gastroenterology. (1996) 111:1125–33. doi: 10.1016/S0016-5085(96)70082-3

49. Tarugi P, Lonardo A, Gabelli C, Sala F, Ballarini G, Cortella I, et al. Phenotypic expression of familial hypobetalipoproteinemia in three kindreds with mutations of apolipoprotein B gene. J Lipid Res. (2001) 42:1552–61. doi: 10.1016/S0022-2275(20)32208-2

50. Wagner RD, Krul ES, Tang J, Parhofer KG, Garlock K, Talmud P, et al. ApoB-54.8, a truncated apolipoprotein found primarily in VLDL, is associated with a nonsense mutation in the apoB gene and hypobetalipoproteinemia. J Lipid Res. (1991) 32:1001 11. doi: 10.1016/S0022-2275(20)41 997-2

51. Welty FK,Hubl ST, Pierotti VR, Young SG. A truncated species of apolipoprotein B (B67) in a kindred with familial hypobetalipoproteinemia. J Clin Invest. (1991) 87:1748–54. doi: 10.1172/JCI115193

52. Whitfield AJ, Marais AD, Robertson K, Barrett PH, van Bockxmeer FM, Burnett JR. Four novel mutations in APOB causing heterozygous and homozygous familial hypobetalipoproteinemia. Hum Mut. (2003) 22:178. doi: 10.1002/humu. 9163
